# Association of Cesarean Delivery and Formula Supplementation with the Stool Metabolome of 6-Week-Old Infants

**DOI:** 10.3390/metabo11100702

**Published:** 2021-10-13

**Authors:** Anne G. Hoen, Modupe O. Coker, Juliette C. Madan, Wimal Pathmasiri, Susan McRitchie, Erika F. Dade, Brett T. Doherty, Susan Sumner, Margaret R. Karagas

**Affiliations:** 1 Department of Epidemiology, The Geisel School of Medicine at Dartmouth, Hanover, NH 03755, USA; mc2190@sdm.rutgers.edu (M.O.C.); juliette.madan@dartmouth.edu (J.C.M.); Erika.F.Dade@dartmouth.edu (E.F.D.); bdoherty@healthcore.com (B.T.D.); Margaret.R.Karagas@dartmouth.edu (M.R.K.); 2 Center for Molecular Epidemiology, The Geisel School of Medicine at Dartmouth, Hanover, NH 03755, USA; 3 Department of Oral Biology, Rutgers School of Dental Medicine, Newark, NJ 07103, USA; 4 Departments of Pediatrics and Psychiatry, Children’s Hospital at Dartmouth, Lebanon, NH 03766, USA; 5 Department of Nutrition, Nutrition Research Institute, University of North Carolina at Chapel Hill, Chapel Hill, NC 27599, USA; susan_mcritchie@unc.edu (S.M.); susan_sumner@unc.edu (S.S.)

**Keywords:** breastfeeding, delivery mode, Cesarean section, infancy, fecal metabolome

## Abstract

Cesarean delivery and formula feeding have both been implicated as important factors associated with perturbations to the infant gut microbiome. To investigate the functional metabolic response of the infant gut microbial milieu to these factors, we profiled the stool metabolomes of 121 infants from a US pregnancy cohort study at approximately 6 weeks of life and evaluated associations with delivery mode and feeding method. Multivariate analysis of six-week stool metabolomic profiles indicated discrimination by both delivery mode and diet. For diet, exclusively breast-fed infants exhibited metabolomic profiles that were distinct from both exclusively formula-fed and combination-fed infants, which were relatively more similar to each other in metabolomic profile. We also identified individual metabolites that were important for differentiating delivery mode groups and feeding groups and metabolic pathways related to delivery mode and feeding type. We conclude based on previous work and this current study that the microbial communities colonizing the gastrointestinal tracts of infants are not only taxonomically, but also functionally distinct when compared according to delivery mode and feeding groups. Further, different sets of metabolites and metabolic pathways define delivery mode and diet metabotypes.

## 1. Introduction

Diverse microbial communities colonize the intestinal tracts of newborns within hours to days of life [1], ultimately serving their host with critical physiological functions including nutrient and drug metabolism, immune maturation, and regulation of inflammatory processes [2,3,4,5,6,7,8,9]. The early life exposures governing the initial assembly of the gut-associated microbiota in infancy have been recently examined using both 16S rRNA gene and shotgun metagenomic sequencing methods, and it is now well established that two important factors shaping the composition and genomic makeup of the infant gut microbiome are delivery mode (vaginal vs. Cesarean section delivery) and feeding pattern (breast milk vs. formula feeding) [10,11,12,13,14,15,16].

Stool metabolomic profiling offers a view of the final products of complex cellular regulation in the gut and can be considered a functional readout of gut microbiota-diet-host metabolism [17,18] that offers a view distinct from that of sequence-based platforms. The microbiota in the human gut is exceptionally diverse, with a long evolutionary history of living in communities with each other and in association with their human host and a propensity to evolve through horizontal gene transfer [19]. Subtle strain-level variation can result in two taxonomically similar communities performing different functions. In addition, sequence-based characterizations of microbial communities are not sensitive to functional redundancy of microbial communities, wherein multiple evolutionarily divergent bacterial taxa with different ecological niches contribute the same function to the collective phenotype of a microbial community. Finally, microbe–microbe interactions and microbe–host interactions may influence the regulation of complex biochemical pathways in the microbial communities of the intestinal tract in ways that cannot be predicted even from detailed metagenomic sequence data. Metabolomics provides a physiologically meaningful and powerful signal of the microbiome’s functional activity or phenotype; however, the normal infant fecal metabolome and any consequences of delivery mode and early feeding patterns on its development have not been described.

In this study, we profiled the stool metabolomes of 121 infants from a US pregnancy cohort study at approximately 6 weeks of life and evaluated associations with delivery mode and feeding method. We used ^1^H NMR analysis to perform both broad-spectrum profiling of metabolomic features and to measure the relative concentrations of 37 known microbial-related metabolites in the stool. Previously in this cohort, we applied 16S rRNA gene sequence-based taxonomic profiling to stool samples collected from 6-week-old infants [10]. We observed that stool microbiome composition was associated with both delivery mode and feeding method and that infants fed a diet of both formula and breast milk had a stool microbiome that resembled that of infants exclusively fed formula. Specifically, our study and others have found that Cesarean delivery and formula feeding are both associated with a lower relative abundance of *Bifidobacteria* and *Bacteroides*, two common infant gut bacterial genera that produce short-chain fatty acids (SCFAs) during carbohydrate fermentation. We therefore tested the hypotheses that delivery mode and feeding method would be similarly associated with the composition of the infant stool metabolome, including with perturbations to SCFA concentrations in stool. This work contributes evidence that feeding and delivery mode, in addition to shaping the composition of the infant gut microbiome, also govern its functional character.

## 2. Results

### 2.1. Participant Characteristics

**Figure 1:**
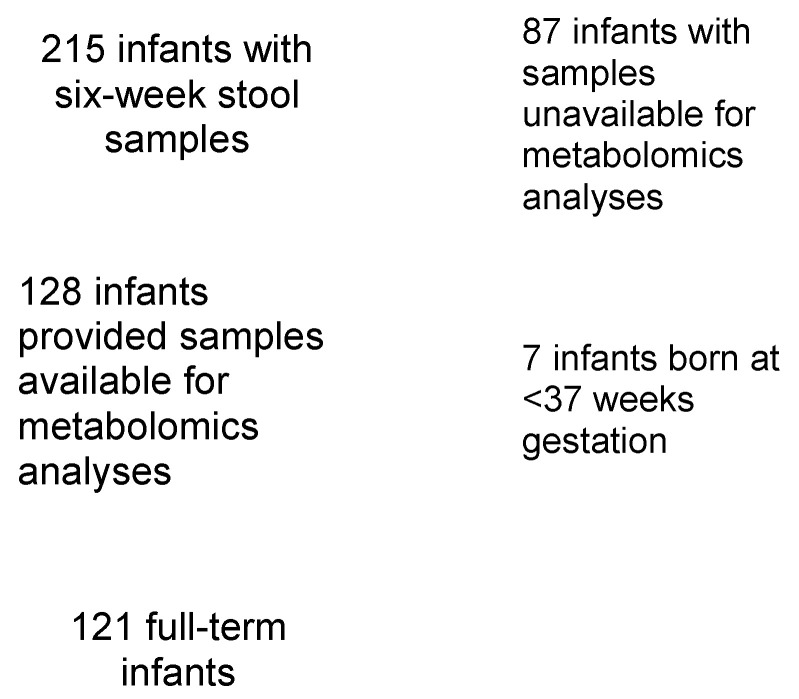
Flow diagram: 215 infants enrolled in the New Hampshire Birth Cohort Study provided a 6-week stool sample. Infants from this group were removed from the current study either because their stool sample was not available for metabolomics analysis (*n* = 87) or because they were born prematurely (*n* = 7), leaving 121 subjects for inclusion in the present study.

We evaluated associations between stool metabolomics profiles and both delivery mode and feeding method in 6-week-old infants enrolled in the New Hampshire Birth Cohort Study (NHBCS). The NHBCS is a pregnancy cohort enrolling mothers in the second trimester of pregnancy, with enrollment and follow-up ongoing. Infants born to enrolled mothers were included in the present study if their mothers provided an infant stool sample collected at approximately 6 [mean (range); 7 (3–22)] weeks of age, authorized the release of the delivery medical record, and responded to questionnaires on feeding practices. Because we aimed to evaluate the metabolomic profiles in association with delivery and feeding modes during normal development, we excluded from our main analysis infants born before 37 weeks gestational age (Figure 1). Delivery medical records and questionnaires were used to assess participant characteristics including delivery mode and feeding method up to the time of stool sample collection in 121 subjects (Table 1).

### 2.2. Multivariate Associations between Metabolomic Profiles and Feeding and Delivery Modes

**Figure 2:**
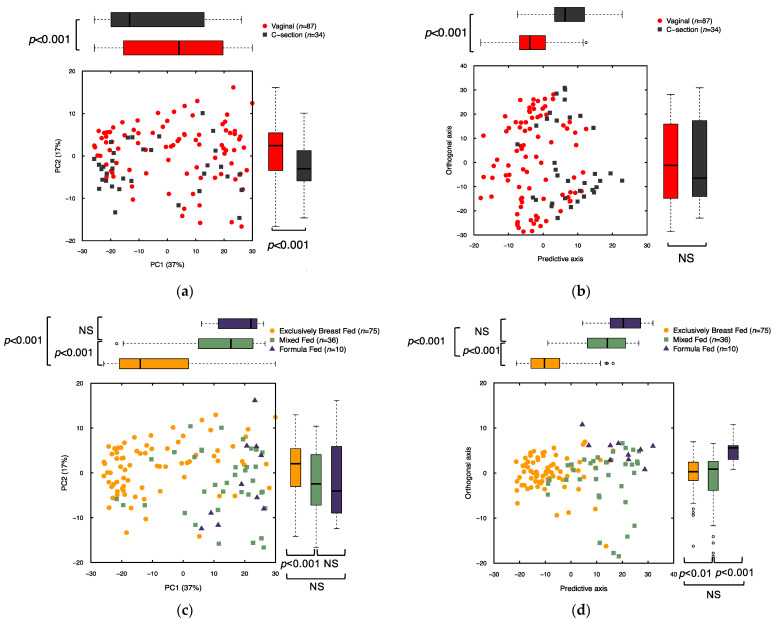
Comparison of stool metabolome between delivery mode and feeding method groups for *n* = 121 subjects. (**a**) PCA of binned NMR data colored by delivery mode. Number of components = 15, Model statistics: R^2^X = 0.9, Q^2^ (cum) = 0.46; (**b**) OPLS-DA of binned NMR data colored by delivery mode. Number of Components = 2 (1 predictive, 1 orthogonal), Model statistics: R^2^X = 0.55, R^2^Y = 0.31, Q^2^ (cum) = 0.14; (**c**) PCA of binned NMR data colored by feeding type. Number of components = 15, Model statistics: R^2^X = 0.9, Q^2^ (cum) = 0.46; (**d**) OPLS-DA of binned NMR data by feeding type. Number of components = 3 (2 predictive, 1 orthogonal), Model statistics: R^2^X = 0.58, R^2^Y = 0.39, Q^2^ (cum) = 0.18. Individual subjects are represented by points marked according to delivery mode (A and B) or feeding type (C and D). Box plots compare groups along each axis, with the heavy black line indicating the group’s median value, box representing the interquartile range, and whiskers extend to the most extreme point, which is no more than 1.5× the interquartile range from the end of the box. *p*-values indicate significant differences between groups along individual axes according to Student’s *t*-test (for 2 independent groups) or ANOVA (for >2 groups and pairwise comparisons; NS indicates not significant).

Binning of NMR spectra resulted in 208 bins that were integrated and normalized to produce relative metabolite signals. Binned data were analyzed by both unsupervised principal component analysis (PCA) and supervised orthogonal partial least squares discriminant analysis (OPLS-DA) to examine whether a metabolic profile could be found in the broad-spectrum metabolomics data that differentiated the study phenotypes (delivery mode and feeding groups) (Figure 2). Using these multivariate approaches, we examined associations along each of the first 2 model components for the delivery mode models, i.e., vaginal vs. Cesarean section delivery (PCA of binned data, R^2^X = 0.9, Q^2^ (cum) = 0.46; *p* < 0.001 for first 2 components; OPLS-DA, 1 predictive and 1 orthogonal component, R^2^X = 0.55, R^2^Y = 0.31, Q^2^ (cum) = 0.14, predictive: *p* < 0.001, orthogonal: *p* = 1.00; Figure 2, parts A and B; Table S1). To further investigate this pattern, multivariate associations between metabolomic profile and delivery mode were also analyzed by breaking delivery mode groups into the following sub-groups: spontaneous vaginal, induced vaginal and vaginal delivery after previous Cesarean section, emergency Cesarean section, and elective Cesarean section delivery (Figure S1). Because no significant associations were observed between metabolomic profiles and delivery sub-groups (e.g., emergency versus elective Cesarean section), further analyses only considered vaginal vs. Cesarean section delivery groups.

Associations between metabolomic spectral bin profiles and feeding pattern were evaluated by comparing samples from infants who, from birth until the time of stool sample collection were exclusively breast fed (EBF), exclusively formula fed (EFF), or fed breast milk supplemented with formula (combination fed; CF). Using both PCA and OPLS-DA, we observed that being EBF was associated with stool metabolomic profiles that were distinct from profiles of infants who were either EFF or CF. There was a statistically significant difference between the metabolomic profiles of EBF infants and both EFF and CF infants, but no significant difference between the metabolomic profiles of EFF and CF infants (PCA of binned data by feeding type, R^2^X = 0.9, Q^2^ (cum) = 0.46, EBF vs. CF: *p* < 0.001, EBF vs. EFF: *p* < 0.001 and EFF vs. CF: *p* = 0.14 for the 1st 2 components; OPLS-DA of binned data by feeding type, 2 predictive and 1 orthogonal component, R^2^X = 0.58, R^2^Y = 0.39, Q^2^ (cum) = 0.18, EBF vs. CF: *p* < 0.001, EBF vs. EFF: *p* < 0.001 and EFF vs. CF: *p* = 0.23 for both orthogonal and predictive components) (Figure 2, parts C and D). Based on these patterns, two feeding status groups were used in the downstream analyses: EBF infants were compared with formula-fed (FF) infants, which included both EFF and CF infants (Figure S2).

In the sensitivity analyses, we repeated these multivariate analyses on a series of subsets of subjects defined according to receipt of intrapartum and postpartum antibiotics and infant sex (Table S1). Our results were robust to the exclusion of infants who received antibiotics. We failed to identify the same associations between metabolomic profiles and delivery mode among the 67 male babies that we observed in the full sample of 121 infants even while qualitatively similar patterns were observed among the 54 female babies. Associations with feeding method remained even among the group of 34 Cesarean-born infants. Form of anesthesia (epidural vs. general) did not appear to impact the metabolome among the Cesarean-born infants, and adjustment for maternal BMI and smoking did not qualitatively change the observed patterns of group associations. Finally, we aimed to address the possibility that some combination-fed infants may have been breast fed a few times but were nearly exclusively breast fed, and that this might have been the driver of the metabolomic profile difference we observed between EBF and CF infants. We identified 11 CF infants who were fed breast milk for less than 2 weeks and reassigned them to the EFF group and then repeated the multivariable analysis (OPLS-DA), and while the model statistics were less predictive, qualitatively there were no differences in the results; therefore, we proceeded with feeding groups as previously defined (data not shown).

Finally, we were interested in examining the joint effect of delivery and feeding on stool metabolome composition. To do this, we performed an additional multivariate analysis examining metabolomic profiles for groups of infants defined jointly by delivery and feeding (vaginally delivered and EBF; Caesarean delivered and EBF; vaginally delivered and FF; and Caesarean delivered and FF). We observed that feeding method was a stronger predictor of metabolomic composition than delivery mode (Figure S3).

### 2.3. Library Matching of NMR Bins

NMR bins with variable importance on projection (VIP) scores ≥ 1 and a jack-knife confidence interval that did not include 0 in OPLS-DA were considered important for distinguishing groups [20] and were library matched to metabolites. A total of 32 bins were identified as important for distinguishing vaginally delivered infants from Cesarean-delivered infants according to VIP scores (Table S2). Fucose, galactose, glucose, arabinose, threonine, alanine, and glycerol were among the 15 compounds identified as library-matched metabolites that were observed in significantly (*p* ≤ 0.05) higher levels in the stool of Cesarean section-delivered infants compared with vaginally delivered, while succinate, glutamate, glucose, methionine, alanine, lysine, lactate, valine, and propionate were among the 17 metabolites found in lower levels in the stool of Cesarean section-delivered infants compared with vaginally delivered. None of these associations were significant (q < 0.1) after controlling for the false discovery rate (FDR) to account for multiple hypothesis testing. For feeding mode, 49 bins were identified as important for separating samples according to infant feeding pattern with VIP scores ≥1 and FDR adjusted *p*-values (q) < 0.1 (Table S3). Propylene glycol, fucose, threonine, glutamate, valine, and alanine were among the 20 metabolites observed in higher levels in the stool of infants fed any formula until the time of sample collection compared with infants fed only breast milk, while malonate, butyrate, lysine, leucine, and propionate were among 29 found in lower levels in the stool of infants fed any formula until the time of sample collection compared with infants fed only breast milk.

### 2.4. Associations between Concentrations of Specific Microbially Derived Metabolites and Delivery and Feeding Modes

**Figure 3:**
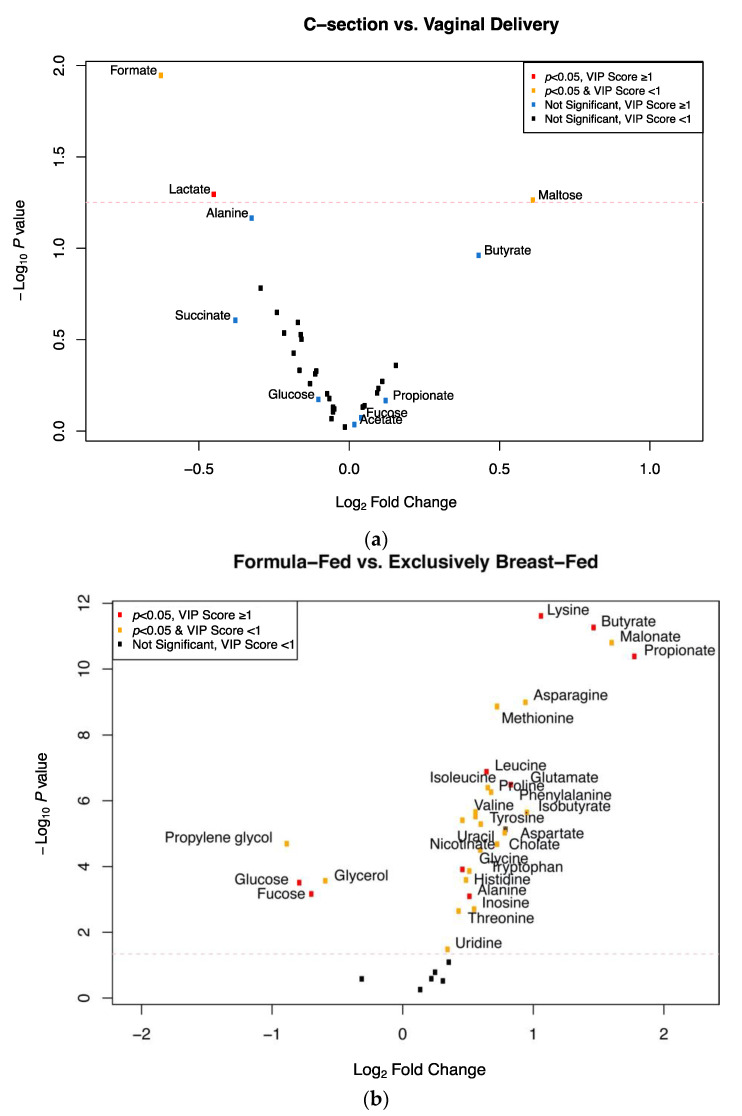
Associations between relative concentrations of metabolites and delivery mode and feeding method. (**a**) Delivery mode. Positive log_2_ fold change values indicate associations with Cesarean section delivery while negative log_2_ fold change values indicate associations with vaginal delivery. (**b**) Feeding method. Positive log_2_ fold change values indicate associations with formula feeding (combination fed and exclusively formula fed) while negative log_2_ fold change values indicate associations with exclusive breast feeding.

Based on a curated metabolite list from the literature [21,22,23], we manually library matched NMR signals to metabolites and relative concentrations were determined using the Chenomx library for a set of 37 co-metabolic products [21,22,23] associated with human gut-dwelling microbes in individual spectra from each of the 121 samples in this study to evaluate associations between the relative abundance of metabolites in infant stool and delivery mode and feeding group (Table 2 and Table 3, Figure 3). Levels of formate (log_2_ fold change = −0.62, SE = 0.24, *p* = 0.006) and lactate (log_2_ fold change = −0.45, SE = 0.22, *p* = 0.008) were significantly lower in stool samples from Cesarean section-delivered infants compared to vaginally delivered infants, while maltose (log_2_ fold change = 0.61, SE = 0.31, *p* = 0.02) was significantly more enriched in stool samples from Cesarean section-delivered infants compared with vaginally delivered infants. As observed with the binned data, none of these metabolites reached a significant FDR threshold (q < 0.1; Table 2). With respect to feeding, propionate (log_2_ fold change = 1.78, SE = 0.24, *p* < 0.001), malonate (log_2_ fold change = 1.61, SE = 0.22, *p* < 0.001), butyrate (log_2_ fold change = 1.47, SE = 0.19, *p* < 0.001), and lysine (log_2_ fold change = 1.07, SE = 0.14, *p* < 0.001) were among the 25 compounds significantly higher in FF infants compared to EBF infants. Glycerol (log_2_ fold change = 0.59, SE = 0.16 *p* < 0.001), fucose (log_2_ fold change = 0.69, SE = 0.20, *p* = 0.001), glucose (log_2_ fold change = 0.78, SE = 0.21, *p* < 0.001), and propylene glycol (log_2_ fold change = 0.88, SE = 0.20, *p* < 0.001) were enriched in EBF infants compared with FF infants. All of these metabolites reached a significant FDR threshold (q < 0.1; Table 3).

### 2.5. Pathway Analysis

**Figure 4:**
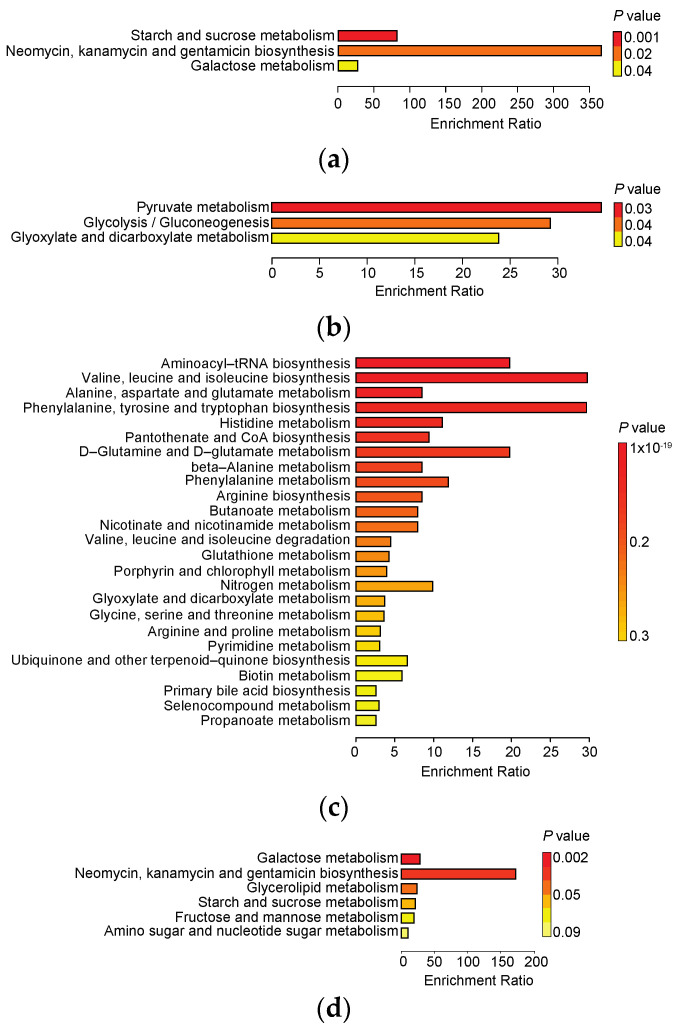
Summary plots of results from metabolite set enrichment analysis. Over representation analysis showed metabolite sets that were represented more among (**a**) Caesarean-delivered, (**b**) vaginally delivered, (**c**) formula-fed, and (**d**) exclusively breast-fed infants.

Finally, we interrogated all 32 metabolites that were associated with either delivery mode (*n* = 3; Table 2) or feeding pattern (*n* = 29; Table 3) at a significance threshold (of a nominal *p* value of ≤0.05) to find perturbations in metabolic pathways (Kyoto Encyclopedia of Genes and Genomes (KEGG)) upon delivery mode or feeding type. Starch and sucrose metabolism pathways were frequently found to be enriched in stool samples from Cesarean section-delivered infants compared with vaginally delivered infants (Figure 4, part A). Pyruvate metabolism, glycolysis/gluconeogenesis, and glyoxylate and dicarboxylate metabolism pathways were found to be enriched in stool samples of vaginally delivered infants compared with Cesarean section-delivered infants (Figure 4, part B). Meanwhile, several amino acid metabolism and biosynthesis pathways, among others, were enriched following formula feeding compared with exclusive breastfeeding, and sugar and lipid metabolism pathways were enriched in infants fed breast milk exclusively compared with those fed any formula (Figure 4, parts C and D).

## 3. Discussion

The aim of this study was to characterize the association between infant gut metabolome, delivery mode, and infant feeding method. We performed previous work in this cohort evaluating microbiome taxonomic profiles between the same delivery and feeding groups in a subset of the same stool samples collected at approximately 6 weeks of age [10]. In that study, we observed significant associations between microbiome composition and both delivery mode and feeding method. Here, we observed associations between overall metabolomic profiles and delivery mode and feeding pattern groups that qualitatively mirror those results. Interestingly, consistent with our previous work that showed the microbiome composition was not significantly different between infants fed formula exclusively compared with those on some form of formula supplementation [10], our current study did not find a metabolic profile that differentiated babies fed formula exclusively from babies given formula as supplement to breast milk. However, we did find significant differences between both groups receiving formula (exclusively or in combination with breast milk) when compared with those fed breast milk exclusively in both studies. Our previous comparison of the relative impacts of delivery mode and feeding method on shaping the infant gut microbiome composition at 6 weeks of age suggested that delivery mode had a stronger effect than feeding method. In the present study, feeding was associated with a greater number of differences in both metabolite concentration and their represented pathways than delivery. This is evidence that while both feeding method and delivery mode are important determinants of infant gut microbiome taxonomic composition and its resulting metabolome, feeding mode may shape the functional character of the gut microbiome in ways that are not apparent with taxonomic profiling. This is interesting in light of our recent detailed interrogation of taxa–function relationships in this cohort, which revealed that infant stool microbial taxonomic and metabolomic profiles were broadly correlated but that microbial relative abundance were not a good predictor of metabolite concentration [24].

Few studies have previously examined the relationships between the stool metabolome and delivery mode and feeding in full-term infants. A study of 77 Chinese infants by Li and colleagues also found differences between feeding groups in the stool metabolomic profiles, including enriched fatty acid biosynthesis pathways among formula-fed infants when compared to exclusively breast fed infants [25]. Li and colleagues did observe differences in metabolomic profiles between infants who were fed formula exclusively vs. in combination with breast milk; however, infants in the study ranged from 2 to 26 weeks of age at the time of stool collection. A study by Bridgman and colleagues of 163 Canadian infants aged 3 to 5 months and a study by Bazanella and colleagues of 106 German infants found that concentrations of SCFAs varied between feeding groups [26,27]. Like both studies, we observed higher concentrations of butyrate and propionate, and as in the Canadian study, higher concentrations of isobutyrate among infants fed formula compared with those fed breast milk. Another study of US infants also found higher concentrations of isobutyrate and propionate but lower concentrations of butyrate in infants fed formula compared with those fed breast milk [28]. Breastfeeding is associated with an increased relative abundance of *Bifidobacterium spp.* in the infant gut [27,29,30]. While bifidobacteria are known to produce lactate and acetate, which can be converted to butyrate by other fecal bacteria, it does not produce butyrate [29]. We also observed an association between formula feeding and enrichment for amino acids and metabolites linked to several amino acid metabolism pathways. This result is aligned with previous observations [30] and could be a consequence of formula feeding driving enrichment for protein-digesting bacteria in the infant gut due to its high protein content compared with breast milk [26]. Heavey and colleagues previously found that products of bacterial protein degradation were higher in infants fed formula than in those fed breast milk [31], and a study by Chow and colleagues also found that formula feeding was associated with a metabolomic profile reflective of a carbon-limited environment resulting in protein fermentation [32]. Interestingly, the branch-chain fatty acid isobutyrate, which we found in greater concentration in the stool of FF compared with EBF infants, is an end-product of microbial protein fermentation. On the other hand, we found that breast feeding was associated with increased glucose and fucose as well as metabolites involved in several carbohydrate fermentation pathways in the infant gut compared with FF subjects, which could be reflective of bacterial fermentation of complex carbohydrates, such as human milk oligosaccharides, in the breast-fed infant gut [30].

Fewer studies have addressed differences in infant fecal metabolomic profiles according to delivery mode, but the study by Bazanella et al. did not find differences by delivery mode groups. We observed elevated concentrations of maltose in Cesarean section-delivered infants compared with vaginally delivered infants in our study, a finding that was also made in a recent study of 60 Chinese infants [33]. It is difficult to speculate on the causal underpinnings of the differences in the concentration of the few metabolites found to be associated with delivery mode. However, it is somewhat surprising that the differences are not more extensive considering that delivery is robustly associated with major differences in microbiota composition in our cohort and in other studies [10,34,35,36,37]. It is conceivable that functional redundancy among bacterial taxa responsible for delivery mode-driven differences in the infant stool microbiome is partly responsible for this pattern.

Infant delivery mode and feeding practices have well-established associations with the infant gut microbiome [10,11,12,15,38,39,40,41,42,43,44,45]; however, the processes relating perturbations to the infant gut microbiome by early life factors to downstream health effects are only beginning to be discovered. Because the host–microbe interactions relevant to human health are frequently mediated by the small molecules secreted, degraded, and/or modified by microbial metabolic processes, work toward the development of microbiome-targeted therapeutics and interventions will benefit from viewing the microbiome through both genomic and metabolomic lenses [46]. Building upon our previous research that identified associations between the two important and common early life factors of delivery mode and feeding method and the taxonomic composition of the infant gut microbiome, the results of the current study indicate that these patterns are also reflected in the infant stool metabolome of infants in the same cohort. This is a promising step toward clarifying the mechanistic underpinnings of the early development of the infant gut microbiome, the factors that shape it, and its lifelong health effects.

Limitations of our study include our study population, which was selected from a relatively ethnically and racially homogenous rural US population sampled at one point in time. We were also underpowered to assess the joint effects of delivery mode and feeding on metabolome profiles. While this is, to our knowledge, the largest study evaluating the effects of both delivery mode and infant feeding on the infant stool metabolome, replicating these results in larger, multi-center studies and in longitudinal analyses will be key to generalizability and to achieving the power needed to examine these relationships in more detail.

## 4. Materials and Methods

The New Hampshire Birth Cohort Study (NHBCS) is an ongoing prospective cohort study of women and their offspring. For this study, pregnant women were recruited from New Hampshire prenatal clinics beginning at approximately 24 to 28 weeks gestation as previously described [47]. Women aged 18–45 years with singleton pregnancies and who are not planning to move are eligible for enrollment. One original objective of the NHBCS was to examine the effects of toxic metals in drinking water on maternal and child health, and at the time of collection of the samples used in the present study, the use of a private, unregulated well at home was an additional eligibility criterion. Institutional review board approval was obtained from the Center for the Protection of Human Subjects at Dartmouth, and participants provided written informed consent. All methods were performed in accordance with the relevant guidelines and regulations.

Infant stool samples were collected at home using provided diapers, sealed in a separate polyethylene bag, and frozen in a home freezer or kept chilled until transport. Samples were transported in a cooler with ice packs and brought to the routine 6-week post-partum visit within 24 h of collection. Stool samples remained frozen until processing where they were thawed at 4 °C. Using sterile applicators, 0.5–1 g of stool was aliquoted into trace element-free cryovial tubes and then frozen at −80 °C in a biorepository.

De-identified stool aliquots were shipped on dry ice and immediately stored at −80 °C for metabolomics analysis. Samples were randomized into batches. For each batch, samples were thawed and ~150 mg of stool were transferred to MagNA Lyser tubes after recording the weight. Samples were then homogenized with 50% acetonitrile in water by using the Omni Bead Disruptor (Omni International, Kennesaw, GA, USA). Homogenized samples were centrifuged at 16,000 relative centrifuge force (rcf) and the supernatant was separated into another tube. An aliquot (1000 uL, 100 mg equivalent of fecal mass) was transferred into an Eppendorf tube and lyophilized overnight. The dried extract was reconstituted in 700 uL of NMR master mix (containing 0.2 M phosphate, 0.5 mM DSS-d6, and 0.2% sodium azide), vortexed on a multitube vortexer at speed 5 for 2 min, and centrifuged at 16,000 rcf for 5 min. A 600 µL aliquot of the supernatant was transferred into a pre-labeled 5 mm NMR tube for data acquisition on a 700 MHz spectrometer. Additionally, study pooled samples (created from randomly selected study samples) and batch pooled quality control [48] samples were generated from supernatants of study samples and aliquots of supernatants were dried and reconstituted similar to the study samples described above and used for quality control purposes.

NMR metabolomics analysis followed procedures previously described [49,50,51]. Briefly, ^1^H NMR spectra of feces samples were acquired on a Bruker Avance III 700 MHz NMR spectrometer (Bruker BioSpin GmbH, Rheinstetten, Germany) using a 5 mm cryogenically cooled ATMA inverse probe and ambient temperature of 25 °C. A 1D NOESY pre-saturation pulse sequence (noesygppr1d, [recycle delay, RD]-90°-t1-90°-tm-90°-acquire free induction decay (FID)]) was used for data acquisition. For each sample, 64 transients were collected into 64k data points using a spectral width of 12.02 ppm, 2 s relaxation delay, 10 ms mixing time, and an acquisition time of 3.899 s per FID. The water resonance was suppressed using resonance irradiation during the relaxation delay and mixing time. NMR spectra were processed using TopSpin 3.5 software (Bruker-Biospin, Germany). Spectra were zero filled and Fourier transformed after exponential multiplication with a line broadening factor of 0.5. The phase and baseline of the spectra were manually corrected for each spectrum. Spectra were referenced internally to the DSS-d6 signal. The quality of each NMR spectrum was assessed for the level of noise and alignment of identified markers. Spectra were assessed for missing data and underwent quality checks. NMR bins (0.49–9.0 ppm) were created excluding water (4.73–4.85 ppm) using intelligent bucket integration of a 0.04 ppm bucket width with 50% looseness using ACD Spectrus Processor (ACD Labs Inc, Toronto, ON, Canada). Integrals of each of the bins were normalized to the total integral of each of the spectrum. A representative NMR spectrum is provided in Figure S4.

Chenomx NMR Suite 8.1 Professional software [52] (Chenomx, Edmonton, AB, Canada), which has a library of approximately 350 compounds and a HMDB reference library pack, was used to match the signals in the bins important to differentiating the study groups to metabolites in multivariate data analysis. In addition, the relative concentration of select metabolites related to host-microbial co-metabolism [21,22,23] was determined using the Chenomx NMR Suite 8.1 Professional software [52].

Delivery mode (Cesarean vs. vaginal delivery and, when applicable, indication for Cesarean delivery) was abstracted from maternal delivery records. We evaluated infant diet from birth until the time of stool collection by telephone questionnaires that included questions regarding the duration of breastfeeding and the timing of formula introduction, if any. Infants who were fed breast milk and who had never been given formula prior to the time of stool collection were given the status of exclusive breast milk feeding. Infants who had not been breast fed and who had been fed formula only prior to their stool collection were assigned the status exclusively formula fed. Infants who had received both breast milk and formula prior to their stool collection were identified as having a diet of both breast milk and formula (combination fed).

Model statistics were evaluated to determine the fitness of the models for various subsets of participants (e.g., based on sex, antibiotic use, and method of anesthesia for Cesarean-born infants). Multivariate and statistical analyses were conducted using SIMCA version 14 [20] (Umetrics, Umeå, Sweden) and R version 3.5.2. Normalized binned NMR data were Pareto scaled and mean centered prior to multivariate analysis. Metabolomic profiles were characterized to determine the stool metabolites differentiating the feeding and delivery groups. Unsupervised PCA and supervised OPLS-DA were used to reduce dimensionality and to enable the visualization of the separation of the study groups [53,54,55]. PCA scores plots were inspected to ensure that the QC pool samples were tightly clustered in the center of study samples used to create the pools, a quality control method widely used in metabolomic studies [20,56,57]. The variable influence on projections (VIP) [58] statistics, which summarize the importance of a bin in differentiating the phenotypic groups, were inspected, and bins that had a VIP ≥ 1.0 with a jack-knife 95% confidence interval that did not include 0 were determined to be important for differentiating the study groups. The R^2^Y in OPLSDA models were calculated and interpreted as the proportion of the variance explained by the model and all models used a 7-fold cross-validation to assess the predictive variation of the model (Q^2^).

Student’s *t*-test (for 2 independent groups) or ANOVA (for >2 groups) was used to identify significant differences (*p* value ≤ 0.05) between groups along the first two principal components (PCA) or predictive and orthogonal axes (OPLS-DA). Log_2_ fold differences between groups were calculated and two-tailed Student’s *t*-tests were performed, and the *p*-values adjusted using false discovery rate (FDR) correction. Volcano plots showing significant metabolites or bins by either *p*-value or VIP scores were created. Metabolites with a significant FDR corrected *p*-value, log_2_ fold change, and VIP score ≥ 1 were labeled and colored differently from others. For all analyses, while FDR correction was performed and adjusted *p*-values (q values) presented when appropriate, nominal *p* values ≤ 0.05 were considered significant.

Metabolic set enrichment analyses of differentially expressed metabolites (based on analysis of relative concentration data) were performed using Metaboanalyst software [59] version 5 (http://www.metaboanalyst.ca/MetaboAnalyst/, accessed on 19 May 2021). Over Representation Analysis (ORA) was performed based on a list of significant compounds (*p* ≤ 0.05) as identified by the data. The purpose of this analysis is to determine if one or more metabolite sets were significantly enriched in pathways. Briefly, with a list of compounds loaded into Metaboanalyst^®^, ORA was implemented using a hypergeometric test after compound mapping. Enrichment analysis was performed using metabolite sets based on KEGG human metabolic pathways in MetaboAnalyst^®^.

## Figures and Tables

**Table 1:** Subject characteristics (*n* = 121).

Variable	Mean (Range) or %
Gestational age (weeks)	39.5 (37–43)
Delivery mode	
Vaginal	72%
Spontaneous vaginal	45%
Induced vaginal	25%
Vaginal after Cesarean	2%
Cesarean section	28%
Elective	12%
Emergency	16%
Infant sex	
Male	55%
Female	45%
Infant birth weight (g)	3453 (2490–4710)
Feeding at six weeks	
Exclusively breast fed	60%
Combination feeding	31%
Exclusively formula fed	9%
Duration of breast feeding among combination-fed subjects (weeks)	4.4 (0.4–8.9)
Age at formula introduction among combination-fed subjects (weeks)	2.8 (0.1–8.7)

**Table 2:** Significant (*p* ≤ 0.05) associations between relative concentrations of metabolites and delivery mode.

Metabolite	VIP ^1^	Log_2_ Fold Change ^2^	Unadjusted *p* Value ^3^	FDR *p* Value (q) ^3^
Maltose	0.50	0.61	0.05	0.61
Lactate	1.40	−0.45	0.05	0.61
Formate	0.39	−0.62	0.01	0.40

^1^ VIP: Variable influence on projection from the OPLS-DA model represents that contribution group discrimination. ^2^ Log_2_ fold changes were calculated from group means (comparing the Cesarean-delivered group to the vaginally delivered group). Negative log_2_ fold changes, therefore, reflect a lower concentration in Cesarean-delivered infants compared with those who were delivered vaginally, while the positive log_2_ fold change reflects a higher concentration in Cesarian-delivered infants compared with those delivered vaginally. ^3^ Students T test was used to compare means resulting in unadjusted (nominal) *p* values and the false discovery rate (FDR), also known as the Benjamini-Hochberg corrected, *p* value.

**Table 3:** Significant (*p* ≤ 0.05) associations between relative concentrations of metabolites and feeding types.

Metabolite	VIP ^1^	Log_2_ Fold Change ^2^	Unadjusted *p* Value ^3^	FDR *p* Value (q) ^3^
Propionate	1.94	1.78	<0.001	<0.001
Malonate	0.73	1.61	<0.001	<0.001
Butyrate	1.36	1.47	<0.001	<0.001
Lysine	1.28	1.07	<0.001	<0.001
Isobutyrate	0.13	0.96	<0.001	<0.001
Asparagine	0.52	0.95	<0.001	<0.001
Glutamate	1.61	0.83	<0.001	<0.001
Uracil	0.68	0.79	<0.001	<0.001
Aspartate	0.51	0.79	<0.001	<0.001
Cholate	0.14	0.73	<0.001	<0.001
Methionine	0.64	0.73	<0.001	<0.001
Proline	0.69	0.69	<0.001	<0.001
Isoleucine	0.88	0.66	<0.001	<0.001
Leucine	1.18	0.65	<0.001	<0.001
Tyrosine	0.49	0.61	<0.001	<0.001
Nicotinate	0.14	0.60	<0.001	<0.001
Phenylalanine	0.65	0.57	<0.001	<0.001
Valine	0.96	0.57	<0.001	<0.001
Inosine	0.09	0.55	0.002	0.003
Alanine	1.10	0.52	0.001	0.001
Tryptophan	0.35	0.52	<0.001	<0.001
Histidine	0.34	0.49	<0.001	<0.001
Glycine	1.01	0.47	<0.001	<0.001
Threonine	0.43	0.44	0.002	0.003
Uridine	0.02	0.35	0.033	0.040
Glycerol	0.80	−0.59	<0.001	<0.001
Fucose	1.14	−0.69	0.001	0.001
Glucose	1.66	−0.78	<0.001	<0.001
Propylene glycol	0.66	−0.88	<0.001	<0.001

^1^ VIP: Variable influence on projection from the OPLS-DA model represents that contribution group discrimination. ^2^ Log_2_ fold changes were calculated from group means (comparing the formula-fed group to the exclusively breast-fed group). Negative log_2_ fold changes, therefore, reflect a lower concentration in formula-fed infants compared with those fed exclusively breast milk, while the positive log_2_ fold change reflects a higher concentration in formula-fed infants compared with those fed exclusively breast milk. ^3^ Students T test was used to compare means resulting in unadjusted (nominal) *p* values and the false discovery rate (FDR), also known as the Benjamini-Hochberg corrected, *p* value.

## Data Availability

The metabolomics data presented in this study are openly available in Metabolomics Workbench at https://doi.org/10.21228/M8K69N (accessed on 8 October 2021). Personal characteristics are not publicly available due to human subjects restrictions.

## Supplementary Materials

The following are available online at https://www.mdpi.com/article/10.3390/metabo11100702/s1, Figure S1: Comparison of stool metabolome between specific delivery type groups for n = 121 subjects, Figure S2: Comparison of stool metabolome between feeding method groups collapsed into exclusively breast fed vs. formula fed (any formula) for n = 121 subjects, Figure S3: Joint analysis of delivery and feeding, Figure S4: A representative 700 MHz 1H NMR spectrum of feces extract with some metabolite identifications, Table S1: Model statistics for study subject subgroups, Table S2: Significant NMR bins and library matched metabolites that distinguish Caesarean-delivered vs. vaginally delivered infants (in order of increasing fold change; n = 32 bins), Table S3: Significant NMR bins and library matched metabolites that distinguish formula-fed vs. exclusively breast-fed infants (in order of increasing fold change; n = 49 bins).
