# Supplementary material for: Association of Cesarean Delivery and Formula Supplementation with the Stool Metabolome of 6-Week-Old Infants
Source: Metabolites. 2021 Oct 13;11(10):702. doi: 10.3390/metabo11100702 (PMC8540440; doi:10.3390/metabo11100702)
Supplement: Supplementary file 1 [file metabolites-11-00702-s001.zip › metabolites-1353079-supplementary.pdf]

Table S1. Model statistics for study subject subgroups

Dataset*	PCA					OPLS-DA					
		Delivery mode		Feeding method		Delivery mode			Feeding method		
	Model statistics	Unadjusted *P*	Adjusted† *P*	Unadjusted *P*	Adjusted† *P*	Model statistics	Unadjusted *P*	Adjusted† *P*	Model statistics	Unadjusted *P*	Adjusted† *P*
**1. N=121**	**15 comp<br>R^2X=0.9<br>Q^2=0.46**	**P1; p<0.001<br>P2 ;p<0.001**	**P1; p=0.04<br>P2; p<0.001**	**P1; p<0.001<br>P2; p=0.11**	**P1; p<0.001<br>P2; p=0.14**	1+1<br>**R^2X= 0.55<br>R^2Y=0.31<br>Q^2=0.14**	**Pre p<0.001<br>Ort p=1.00**	**Pre p=0.02<br>Ort p<0.01**	**2+1<br>R^2X= 0.58<br>R^2Y=0.39<br>Q^2=0.18**	**Pre p<0.001<br>Ort p=0.23**	**Pre p<0.001<br>Ort p=0.93**
2. N=119	4 comp<br>R^2X= 0.66<br>Q^2=0.46	P1; p=0.04<br>P2; p<0.001	P1; p=0.05<br>P2; p<0.01	P1; p<0.001<br>P2; p=0.03	P1; p<0.001<br>P2; p=0.04	1+1<br>R^2X= 0.55<br>R^Y=0.31<br>Q2=0.11	Pre p<0.001<br>Ort p=1.00	Pre p<0.001<br>Ort p=1.00	2+1<br>R^2X= 0.58<br>R^2Y=0.39<br>Q^2=0.13	Pre p<0.001<br>Ort p=0.93	Pre p<0.001<br>Ort 0.93
3. N=67	3 comp<br>R^2X= 0.63<br>Q^2=0.42	P1; p=0.95<br>P2; p=0.96	P1; p=1.00<br>P2; p=0.99	P1; p<0.001<br>P2; p=0.71	P1; p<0.001<br>P2; p=0.85	1+1<br>R^2X= 0.29<br>R^2Y=0.16<br>Q^2=-0.21	Pre p<0.001<br>Ort p=1.000	Pre p<0.001<br>Ort p=1.00	2+1<br>R^2X= 0.52<br>R^2Y=0.47<br>Q^2=0.30	Pre p<0.001<br>Ort p=0.99	Pre p<0.001<br>Ort p=1.00
4. N=54	6 comp<br>R^2X= 0.71<br>Q^2=0.54	P1; p=0.16<br>P2; p=0.67	P1; p=0.32<br>P2; p=0.56	P1; p<0.001<br>P2; p=0.21	P1; p<0.001<br>P2; p=0.31	1+1<br>R^2X= 0.44<br>R^2Y=0.43<br>Q^2=-0.02	Pre p<0.001<br>Ort p=1.00	Pre p<0.001<br>Ort p=1.00	2+1<br>R^2X= 0.54<br>R^2Y=0.57<br>Q^2=0.12	Pre p<0.001<br>Ort p=0.93	Pre p<0.001<br>Ort p=0.90
5.‡ N=34	4 comp<br>R^2X= 0.66<br>Q^2=0.47	P1; p=0.49<br>P2; p=0.47	P1; p=0.53<br>P2; p=0.56	P1; p<0.001<br>P2; p=0.21	P1; p<0.001<br>P2; p=0.32	1+1<br>R^2X= 0.64<br>R^2Y=0.72<br>Q^2=0.07	Pre p<0.001<br>Ort p<0.001	Pre p<0.001<br>Ort p<0.001	2+1<br>R^2X= 0.54<br>R^2Y=0.45<br>Q^2=0.30	Pre p<0.001<br>Ort p=0.90	Pre p<0.001<br>Ort p=0.94

*__Dataset 1__: All subjects excluding infants born < 37 weeks (main analysis group); **2:** Dataset 1 excluding infants who received antibiotics (intrapartum and postpartum); **3**: Males of Dataset 1; **4:** Females of Dataset 1; **5:** Cesarean-born infants of Dataset 1 (see note below); comp: components; P1: first principal component; P2: second principal component; PCA: principal components analysis; OPLS-DA: orthogonal partial least squares discriminant analysis; Pre: predictive axis; Ort: orthogonal axis.

P values were derived from linear regression; †Adjusted for maternal smoking and BMI; ‡The column for delivery mode is not applicable here; we instead examine infants born via Cesarean section using epidural anesthesia vs. general anesthesia

Table S2. Significant NMR bins and library matched metabolites that distinguish Caesarean-delivered vs. vaginally delivered infants (in order of increasing fold change; N=32 bins). *These features represent signals that have p value of <0.1 and VIP score of ≥1 (no metabolites were significant at FDR p value (q) of <0.1.*

Bin (ppm)	Library-matched metabolite(s)	VIP score	Log_2 fold change	Unadjusted P value	FDR p value (q)
2.37 .. 2.43	Succinate	2.98	-0.606	0.008	0.12
2.49 .. 2.52	Overlapped	1.40	-0.452	0.013	0.13
2.32 .. 2.37	Glutamate	1.71	-0.395	0.006	0.10
3.39 .. 3.44	Glucose	1.56	-0.361	0.001	0.10
2.60 .. 2.65	Methionine	1.31	-0.358	0.005	0.10
1.44 .. 1.50	Alanine	2.39	-0.358	0.010	0.12
2.98 .. 3.04	Lysine | Creatinine | Creatine	1.86	-0.337	0.051	0.25
1.30 .. 1.36	Lactate | Threonine	3.03	-0.335	0.005	0.10
2.24 .. 2.29	Valine	1.49	-0.322	0.008	0.12
2.20 .. 2.24	Fatty acids	1.18	-0.305	0.058	0.26
1.02 .. 1.07	Propionate | Valine	1.74	-0.300	0.059	0.26
0.97 .. 1.02	Valine | Isoleucine	1.77	-0.259	0.023	0.16
1.95 .. 1.99	Fatty acids	1.30	-0.253	0.027	0.17
1.68 .. 1.74	Leucine | Lysine	1.84	-0.232	0.095	0.33
4.02 .. 4.07	Overlapped	1.01	-0.205	0.004	0.10
2.08 .. 2.14	Butyrate | Glutamate | Glutamine | Methionine	1.43	-0.167	0.069	0.27
4.07 .. 4.13	Lactate | Proline	1.01	-0.097	0.014	0.13
3.81 .. 3.86	Overlapped	3.34	0.163	0.051	0.25
3.71 .. 3.77	Glutamate | Leucine | Lysine | Fucose | Glucose	3.32	0.170	0.045	0.24
3.91 .. 3.96	Overlapped	2.37	0.176	0.072	0.28
4.22 .. 4.27	Threonine | Sugars	1.66	0.190	0.045	0.24
1.25 .. 1.30	Fatty acids	2.12	0.225	0.065	0.26
3.66 .. 3.68	Glycerol | isoleucine	2.52	0.252	0.001	0.10
3.77 .. 3.81	Alanine	3.76	0.257	0.032	0.19
3.86 .. 3.91	Overlapped	3.22	0.258	0.017	0.13
4.39 .. 4.45	Overlapped	1.45	0.288	0.054	0.26
3.68 .. 3.71	Unknown | Glucose	3.73	0.298	0.010	0.12
3.57 .. 3.59	Threonine | Sugars	1.97	0.305	0.031	0.19
4.49 .. 4.55	Arabinose | Fucose	1.13	0.324	0.010	0.12
4.61 .. 4.67	Glucose	1.47	0.334	0.041	0.23
5.26 .. 5.31	Galactose | Arabinose	1.50	0.338	0.073	0.28
1.20 .. 1.22	Fucose | Fatty acids	1.90	0.489	0.003	0.10

Table S3. Significant NMR bins and library matched metabolites that distinguish formula-fed vs. exclusively breast-fed infants (in order of increasing fold change; N=49 bins). *These features represent signals that have q value of <0.1 and VIP score of ≥1.*

Bin (ppm)	Library-matched metabolite(s)	VIP score	Log_2 fold change	Unadjusted P value	FDR p value (q)
3.09 .. 3.15	Malonate | Phenylalanine	1.584	-1.238	<0.0001	4.48E-19
1.50 .. 1.56	Butyrate	2.121	-1.236	<0.0001	6.51E-20
0.86 .. 0.91	Butyrate	2.454	-1.207	<0.0001	1.58E-17
2.14 .. 2.20	Butyrate | Propionate	2.196	-1.094	<0.0001	6.95E-15
2.98 .. 3.04	Lysine | Creatinine | Creatine	2.483	-1.086	<0.0001	1.61E-14
1.74 .. 1.79	Overlapped	1.781	-1.055	<0.0001	7.12E-17
1.42 .. 1.44	Lysine | Isoleucine	1.111	-1.027	<0.0001	6.51E-20
1.58 .. 1.63	Overlapped	1.520	-1.020	<0.0001	7.86E-15
1.02 .. 1.07	Propionate | Valine	2.392	-1.018	<0.0001	2.37E-12
1.63 .. 1.68	Leucine | Lysine	1.790	-0.964	<0.0001	1.82E-14
1.38 .. 1.42	Overlapped	1.308	-0.910	<0.0001	2.01E-14
1.84 .. 1.86	Leucine | Lysine	1.112	-0.905	<0.0001	1.63E-15
1.68 .. 1.74	Leucine | Lysine	2.602	-0.899	<0.0001	3.61E-14
2.20 .. 2.24.	Fatty acids	1.304	-0.867	<0.0001	1.61E-11
3.04 .. 3.09	Tyrosine	1.163	-0.847	<0.0001	5.83E-13
1.92 .. 1.95.	Overlapped	1.112	-0.842	<0.0001	3.45E-17
2.32 .. 2.37	Glutamate	1.809	-0.799	<0.0001	1.52E-10
1.44 .. 1.50	Alanine	2.548	-0.778	<0.0001	4.23E-12
2.43 .. 2.49	Overlapped	1.015	-0.765	<0.0001	1.30E-09
0.91 .. 0.97	Isoleucine | Leucine	2.899	-0.705	<0.0001	5.83E-13
1.86 .. 1.92	Acetate | Lysine | Arginine	3.209	-0.681	<0.0001	3.70E-08
1.95 .. 1.99	Isoleucine | Proline	1.479	-0.644	<0.0001	3.66E-10
2.24 .. 2.29	Valine	1.301	-0.633	<0.0001	2.71E-10
0.97 .. 1.02	Valine | Isoleucine	1.781	-0.571	<0.0001	7.98E-09
2.08 .. 2.14	Butyrate | Glutamate | Glutamine | Methionine	1.748	-0.524	<0.0001	1.17E-10
3.15 .. 3.21	Choline | O-Acetylcholine | O-Acetylcarnitine | Tyrosine	1.020	-0.504	<0.0001	4.93E-08
2.37 .. 2.43	Succinate	1.734	-0.422	0.048	0.0613
4.22 .. 4.27	Threonine | Sugars	1.093	-0.369	<0.0001	9.21E-05
1.30 .. 1.36	Lactate | Threonine	1.144	-0.352	0.001	0.002
3.66 .. 3.68	Glycerol | isoleucine	1.245	0.193	0.006	0.008
3.53 .. 3.57	Glycine | Glycerol	1.607	0.379	<0.0001	3.52E-11
3.68 .. 3.71	Unknown | Glucose	1.181	0.388	<0.001	5.49E-04
3.81 .. 3.86	Overlapped	2.997	0.414	<0.0001	1.41E-07
3.44 .. 3.48	Glucose	1.720	0.488	<0.0001	3.61E-08
3.50 .. 3.53	Glucose	1.472	0.514	<0.0001	1.03E-11
3.62 .. 3.66	Fucose | Glycerol	2.692	0.523	<0.0001	1.11E-10

3.71 .. 3.77	Glutamate | Leucine | Lysine | Fucose | Glucose	4.091	0.535	<0.0001	4.23E-12
3.91 .. 3.96	Overlapped	2.819	0.561	<0.0001	1.11E-10
3.86 .. 3.91	Overlapped	3.291	0.579	<0.0001	9.63E-10
3.48 .. 3.50	Glucose	1.748	0.647	<0.0001	9.87E-11
2.01 .. 2.06	Glutamate | Proline | N-Acetyl amino acids	3.359	0.661	<0.0001	5.83E-13
3.77 .. 3.81	Alanine	3.663	0.682	<0.0001	5.10E-12
3.59 .. 3.62	Valine | Sugars	2.277	0.683	<0.0001	2.05E-14
3.57 .. 3.59	Threonine | Sugars	2.067	0.789	<0.0001	4.23E-12
1.22 .. 1.25	Fucose | Fatty acids	1.408	0.806	<0.0001	1.88E-07
4.39 .. 4.45	Overlapped	1.677	0.852	<0.0001	9.87E-11
4.61 .. 4.67	Glucose	1.069	0.869	<0.0001	2.84E-09
1.18 .. 1.20	Fatty acids	1.699	0.909	<0.0001	3.68E-12
1.12 .. 1.18	Propylene glycol | fatty acids	2.176	0.944	<0.0001	3.61E-14

Figure S1. Comparison of stool metabolome between specific delivery type groups for N=121 subjects. A. Scores plot of PCA of binned NMR data colored by specific delivery type. Number of Components = 15, Model statistics: $R^2Y = 0.82$, $R^2Y = 0.92$, Q^2 (cum) = 0.55; B. Scores plot of OPLS-DA of binned NMR data colored by specific delivery type. Model statistics: Components = 4 predictive, 1 orthogonal, $R^2X = 0.62$, $R^2Y = 0.57$, Q^2 (cum) = -0.0003. Individual subjects are represented by points marked according to specific delivery type. Box plots compare groups along each axis with heavy black line indicating the group's median value, box representing the interquartile range, and whiskers extend to the most extreme point which is no more than 1.5X the interquartile range from the end of the box.

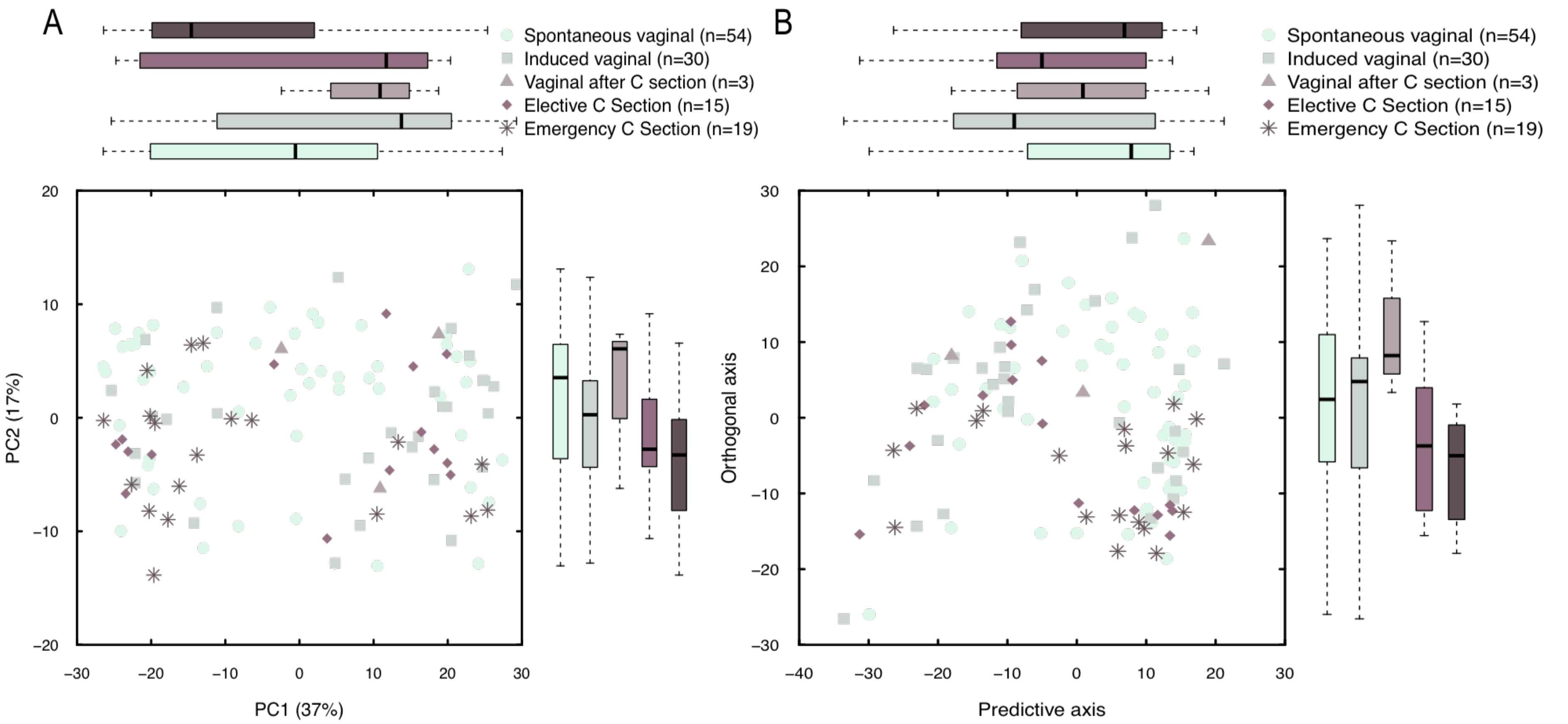

Figure S2. Comparison of stool metabolome between feeding method groups collapsed into exclusively breast fed vs. formula fed (any formula) for N=121 subjects. A. Scores plot of PCA of binned NMR data colored by broad feeding type. Number of components = 15, Model statistics: $R^2X = 0.92$, Q^2 (cum) = 0.52; B. Scores plot of OPLS-DA of binned data by broad feeding type. Number of components = 2 (1 predictive, 1 orthogonal), Model statistics: $R^2X = 0.55$, $R^2Y = 0.52$, Q^2 (cum) = 0.38. Individual subjects are represented by points marked according to feeding method. Box plots compare groups along each axis with heavy black line indicating the group's median value, box representing the interquartile range, and whiskers extend to the most extreme point which is no more than 1.5X the interquartile range from the end of the box. P-values indicate significant differences between groups along individual axes according to Student's t-test (NS indicates not significant).

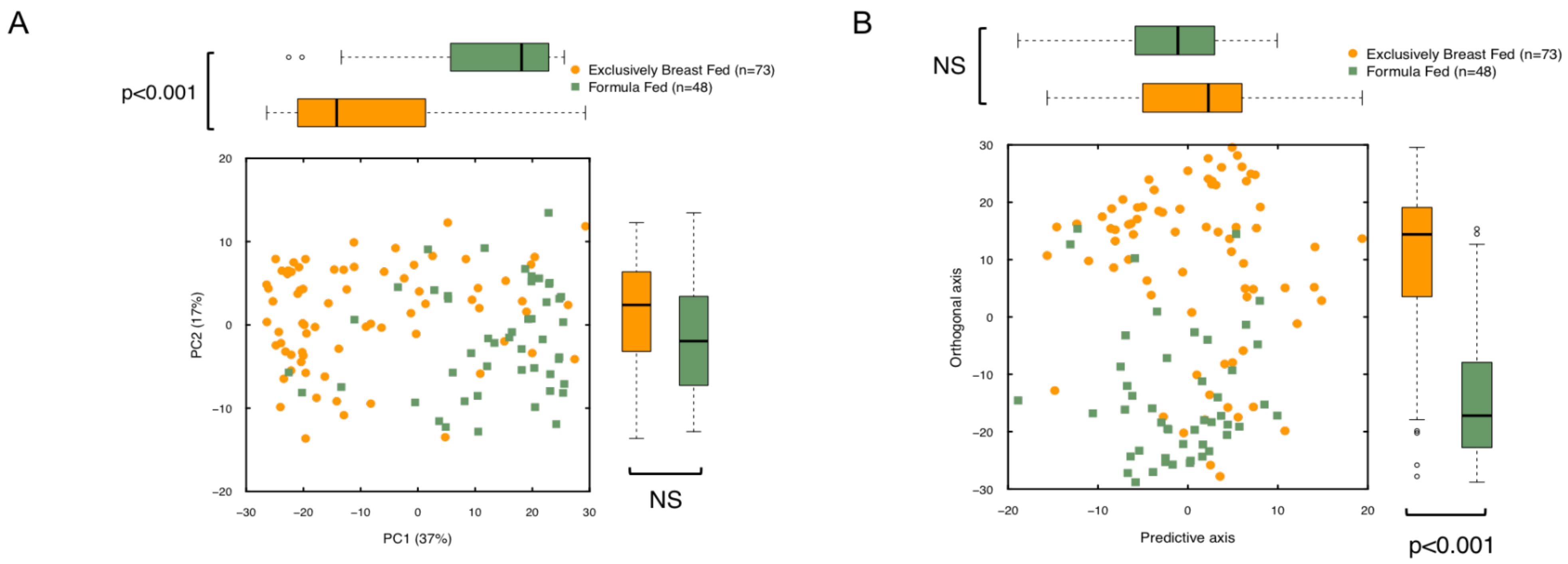

Figure S3. Comparison of stool metabolome between groups based on joint analysis of delivery mode and feeding method for N=121 subjects. A. Scores plot of PCA of binned NMR data colored by the 4 delivery and feeding groups. Number of components = 15, Model statistics: $R^2X = 0.92$, Q^2 (cum) = 0.52; **B.** Scores plot of OPLS-DA of binned data by the combined delivery and feeding groups. Number of components = 2 (3 predictive, 1 orthogonal), Model statistics: $R^2X = 0.60$, $R^2Y = 0.27$, Q^2 (cum) = 0.12. Individual subjects are represented by points marked according to the 4 combined groups. Box plots compare groups along each axis with heavy black line indicating the group's median value, box representing the interquartile range, and whiskers extend to the most extreme point which is no more than 1.5X the interquartile range from the end of the box.

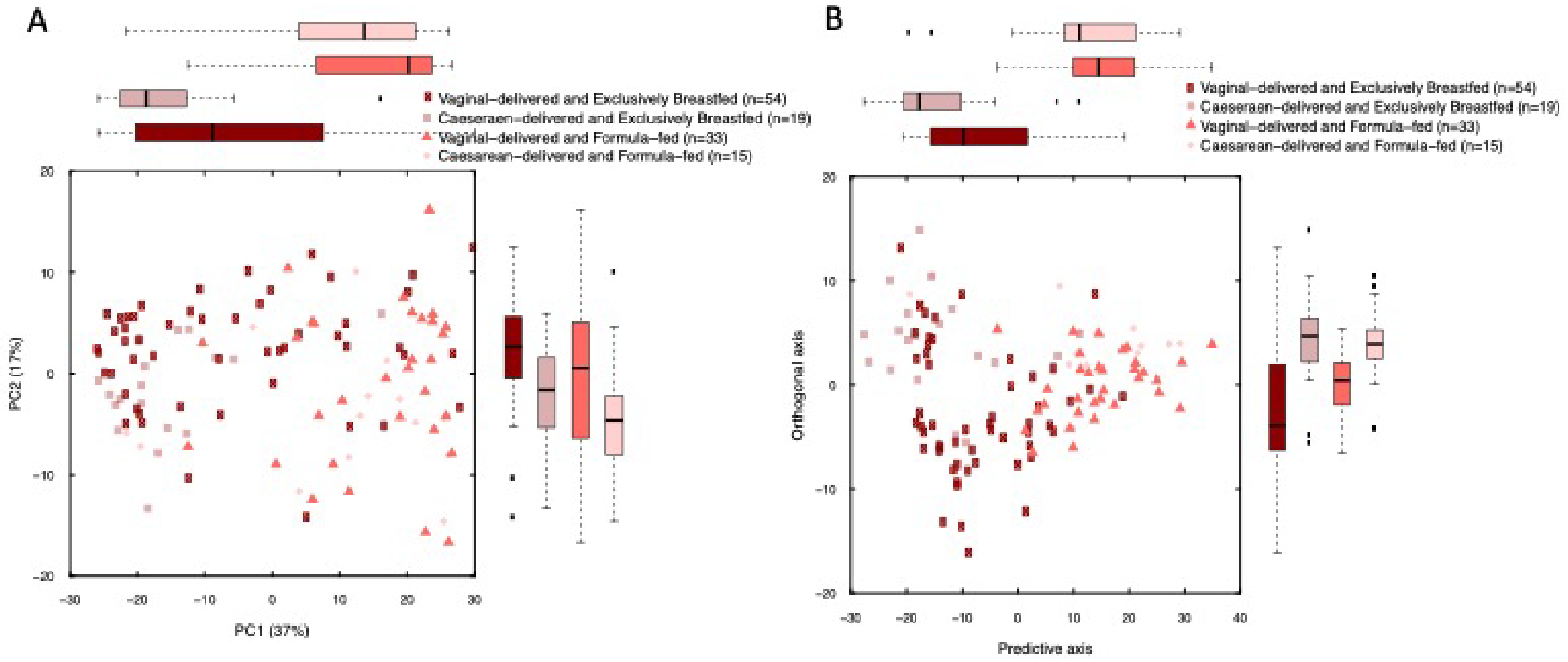

Figure S4. A representative 700 MHz ^{1}H NMR spectrum of feces extract with some metabolite identifications. These metabolites belong to alcohols, amino acids and derivatives, carbohydrates, energy metabolites, carboxylic acids including short chain fatty acids, and purine and pyrimidine metabolites consistent with other published NMR studies involving stool metabolome [1,2]. **A.** Metabolites that are with relatively high abundance in stool samples. The NMR signal labeled as "Unknown" at ~3.7 ppm could not be matched to any metabolite in the Chenomx metabolite library (or HMDB reference library). **B.** Vertically scaled spectrum (in **A**) to show additional identities of metabolites in low abundance in stool samples. Upper panel shows an expanded region (0.82 - 1.14 ppm) showing NMR peaks of short chain fatty acids, butyrate and propionate, and branched chain amino acids, isoleucine, leucine, and valine.

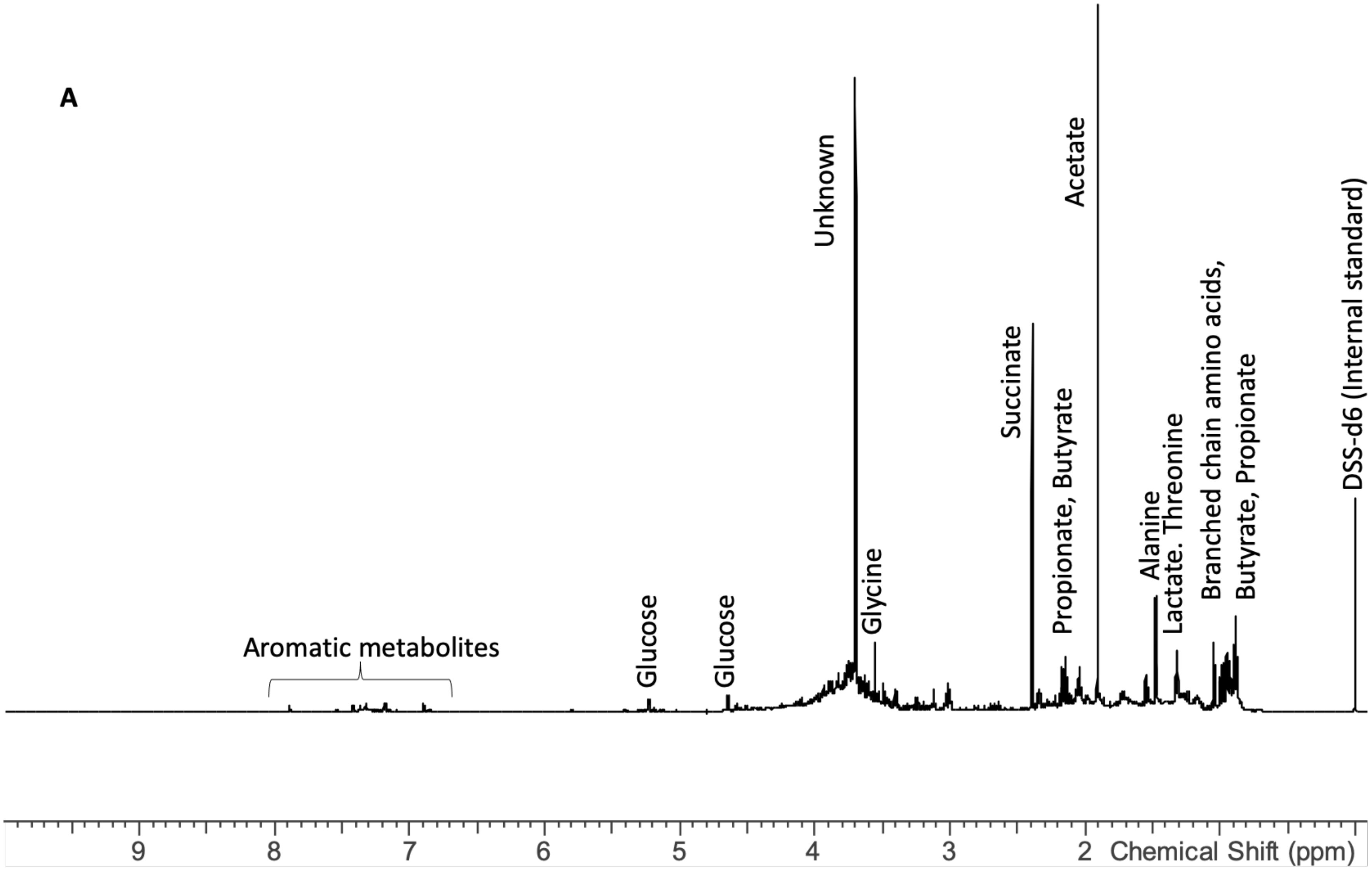

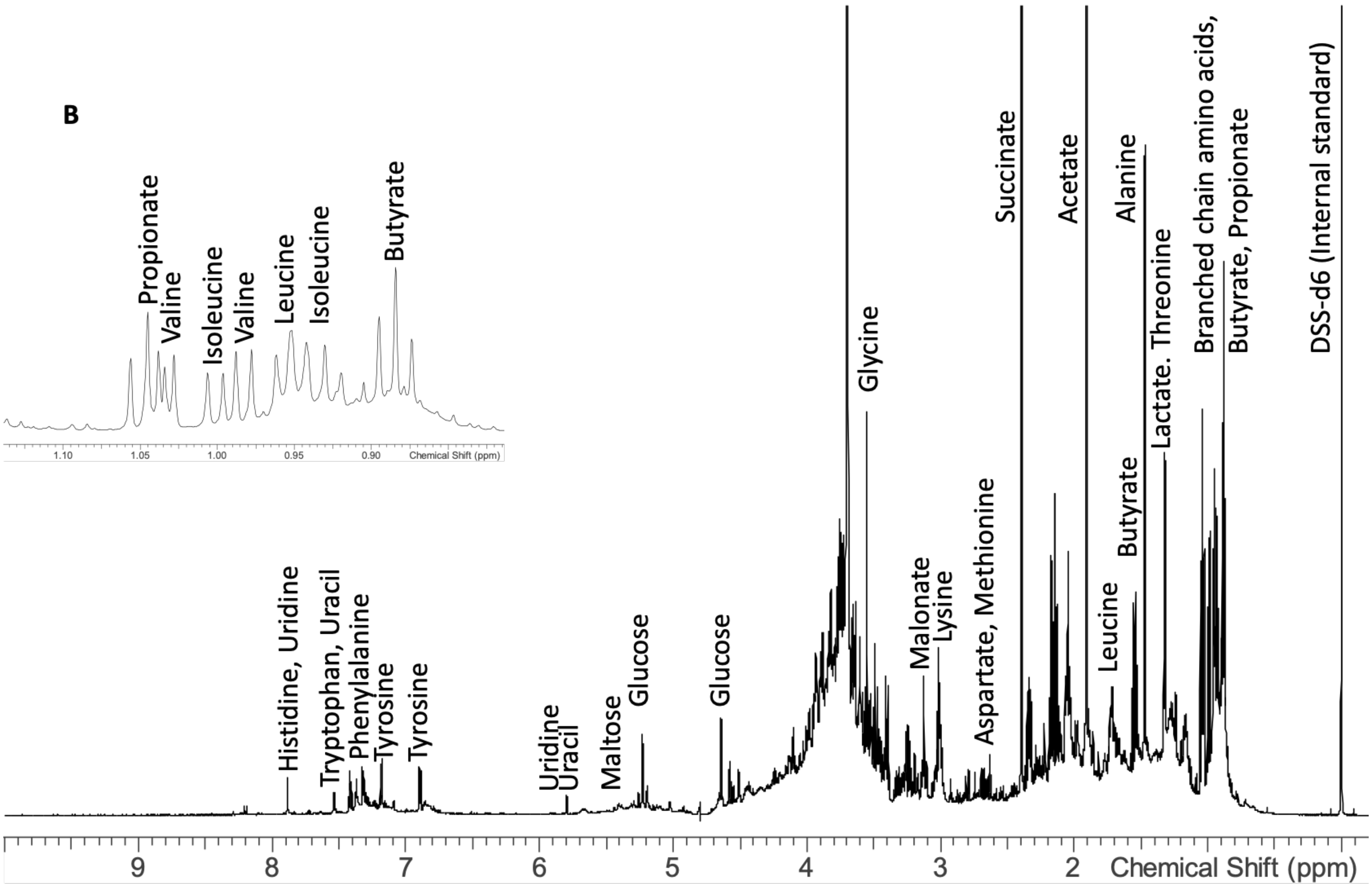

1. Bervoets, L.; Ippel, J.H.; Smolinska, A.; van Best, N.; Savelkoul, P.H.M.; Mommers, M.A.H.; Penders, J. Practical and Robust NMR-Based Metabolic Phenotyping of Gut Health in Early Life. *J Proteome Res* **2021**, doi:10.1021/acs.jproteome.1c00617.
2. Del Chierico, F.; Vernocchi, P.; Petrucca, A.; Paci, P.; Fuentes, S.; Pratico, G.; Capuani, G.; Masotti, A.; Reddel, S.; Russo, A.; et al. Phylogenetic and Metabolic Tracking of Gut Microbiota during Perinatal Development. *PLoS One* **2015**, *10*, e0137347, doi:10.1371/journal.pone.0137347.
